# Change in prey genotype frequency rescues predator from extinction

**DOI:** 10.1098/rsos.220211

**Published:** 2022-06-22

**Authors:** Ruben Joseph Hermann, Lutz Becks

**Affiliations:** Aquatic Ecology and Evolution Group, Limnological Institute University Konstanz, Konstanz, Germany

**Keywords:** predator–prey, indirect evolutionary rescue, rotifer, *Chlamydomonas*, frequency dependency, eco-evolutionary dynamics

## Abstract

Indirect evolutionary rescue (IER) is a mechanism where a non-evolving species is saved from extinction in an otherwise lethal environment by evolution in an interacting species. This process has been described in a predator–prey model, where extinction of the predator is prevented by a shift in the frequency of defended towards undefended prey when reduced predator densities lower selection for defended prey. We test here how increased mortality and the initial frequencies of the prey types affect IER. Combining the analysis of model simulations and experiments with rotifers feeding on algae we show IER in the presence of increased predator mortality. We found that IER was dependent on the ability of the prey to evolve as well as on the frequency of the defended prey. High initial frequencies of defended prey resulted in predator extinction despite the possibility for prey evolution, as the increase in undefended prey was delayed too much to allow predator rescue. This frequency dependency for IER was more pronounced for higher predator mortalities. Our findings can help informing the development of conservation and management strategies that consider evolutionary responses in communities to environmental changes.

## 1. Introduction

Evolutionary rescue (ER) describes the process where a species evolves to avert extinction in an otherwise lethal environment [1,2] through the increase of an adapted genotype with a positive net growth rate. The adapted genotype may be present at low frequency [3], introduced via migration [4,5], the result of genetic mixing [6] or evolve de novo [7,8]. As species interact with other species, it is, however, possible that changes in their interactions and indirect ecological and evolutionary processes affect the demography of a population [9–11] and thus the probability of rescue. Theoretical studies have started to integrate the effect of multi-species interaction into research on ER [12–17]. There are, however, only few studies exploring ER in communities with multiple species [18,19], and the conditions under which species can be rescued through evolutionary change in communities are still poorly explored [20].

Predator–prey interactions are an important driver for population dynamics and intraspecific diversity [9,21] and have been shown to affect species persistence of prey and/or predator through direct and indirect processes [10,22]. Indirect evolutionary rescue (IER) where a species is rescued from extinction through evolution in an interacting species has been suggested as a mechanism promoting predator persistence in an otherwise lethal environment [13]. In their model, Yamamichi & Miner [13] investigated whether heritable trait variation in the prey population determines the interaction with the predator (i.e. defended and undefended prey) and competition between prey clones can rescue the predator from an environmental change that results in increased mortality for the predator. They found that the predator goes extinct with increasing mortality when neither prey nor predator can adapt. However, the predator can survive and gain positive population growth in the presence of increased mortality when the prey species can evolve in response to changes in the predator density. This is because decreased predator density disfavours defended prey and favours more competitive undefended prey, which in return leads to an increase in predator growth and eventually rescue of the predator population. IER in a predator–prey system can lead to an u-shaped temporal change in the predator density following the environmental change similar to ER [1,2], but the change depends on a shift in the frequencies of the defended and undefended prey for IER.

A key assumption for the IER in predator–prey systems with at least two prey clones is that changes in the frequencies of the prey clones can prevent the extinction of the predator when increased predator mortality reduces selection for defended prey and leads to an increase in the frequency of undefended prey. As fitness and thus the persistence of the predator depends on the change in the prey, IER is expected to be a function of the frequency of the defended prey and density of total prey at the time when the environment becomes lethal. For example, when the frequency of the undefended prey is low to begin with, the predator might go extinct before the undefended prey reaches high enough densities. Or when the frequency and density of the undefended prey is initially high, the predator might survive without evolutionary change in the prey. We tested here the frequency dependency of IER by combining the analyses of model simulations and experiments. First we identified combinations of mortality rates of the predator and frequencies of defended prey under which IER occurs by adapting a predator–prey model from Becks *et al.* [21]. In a second step, we tested for IER in experiments using a freshwater predator–prey system consisting of the rotifer *Brachionus calyciflorus* feeding on the green algae *Chlamydomonas reinhardtii*. To manipulate the presence/absence of evolution in the prey species as well as the frequency of defended prey, we used two different isogenic lines of *C. reinhardtii*. The clones differed in their defence trait against the rotifer, where the higher defence correlated with a lower growth rate (electronic supplementary material, figure S1) ([23], for a more detailed description of the traits and correlation between traits see [24]). We used sodium chloride (NaCl) as a stressor because it increases the rotifer mortality but has no significant effect on algal growth at the concentrations considered here (electronic supplementary material, figures S2 and S3). By varying NaCl concentrations, we tested a range of environments in which the rotifer could be rescued through the changes in the frequencies of the two algal clonal lines. Additionally, we manipulated the starting frequencies of the two algal clones to investigate the effect of the populations' defence level on the probability of rescue.

## 2. Material and methods

### 2.1. Model

We used the model from Becks *et al*. [21], which describes the dynamics of a single predator with two different prey clones in chemostats. The prey clones differ in two functional traits: defence against predation and competitiveness. *C_1_* is the undefended clone with a higher palatability (*p_i_*; probability of consumption by the predator) than the defended clone *C_2_*. Investing into higher defences is assumed to lead to a cost of reduced competitiveness, which is modelled here as differences in half-saturation constant (*K_C_*) for the limiting resource *N*, such that *K_C,1_*, of the undefended prey, is smaller than *K_C,2_*, of the defended prey*.* This results in the undefended clone having a higher nutrient uptake at low concentrations. The predator, while consisting of one genotype, is divided into two subpopulations, mimicking our experimental system and following previous studies [21,25]. Reproducing predators (*B*) age with rate *λ* and become predators that are no longer reproducing (*S*) but still consume prey.

2.1 $$\;\frac{dN}{dt}=\delta \;(N_{I}-N)-\frac{\rho C_{1}N}{K_{C,1}+N}-\frac{\rho C_{2}N}{K_{C,2}+N},$$

2.2 $$\frac{dC_{i}}{dt}=C_{i}\;\left[\frac{X_{C}\rho N}{K_{C,i}+N}-\frac{\;p_{i}G(B+S)}{K_{B}+Q}-\;\delta \right]\;i=1,2,$$

2.3 $$\frac{dB}{dt}=B\;\left[\frac{X_{B}GQ}{K_{B}+Q}-(\delta +m+\;\lambda )\right]$$

2.4 $$\;\mathrm{and}\;\;\frac{dS}{dt}=\lambda B-(\delta +m)S.$$

We assume nitrogen (*N*) as limiting resource, which is renewed by a constant inflow with the dilution rate *δ* and at the concentration *N_I_*, ensuring that the resources are not exhausted. Prey population growth and prey nitrogen consumption is modelled as a Monod function with the same nitrogen conversion efficiency *X_C_* and maximum per capita recruitment *β_C_* for both clones included through the parameter *ρ* = *β_C_*/*X_C_*. The nutrient uptake of the prey is modelled to be limited by the half-saturation constants *K_C,1_* = 2 and *K_C,2_* = 8. Both prey clones are grazed by predators following a Monod equation which is influenced by their palatability. The higher palatability of the undefended clone (*p_1_* = 0.8) leads to a higher grazing compared with the defended clone (*p_2_* = 0.2). This leads to a dynamic where the undefended clone is selected for at low and the defended at high predator densities. The grazing parameter *G* = *β_B_/K_B_* is defined by the predator conversion efficiency *β_B_* and predator half-saturation constant *K_B_*. Predator consumption is limited by the total available prey *Q* = *p_1_C_1_* + *p_2_C_2_*. Thus, predator growth is a function of prey densities and the frequencies of the two clones. Reproducing *(B)* and non-reproducing predators *(S)* die at a constant daily mortality rate *m* (all parameters and values table 1).

**Table 1.** Parameters for predator–prey model.

parameter	description	value
*N_I_*	limiting resource	80–370 µM
*δ*	dilution rate	0.1–0.7 d^−1^
*m*	predator mortality	0.055–0.825
*λ*	predator senescence rate	0.4 d^−1^
*X_C_*	prey conversion (10^9^ cells/µmol N)	0.0027
*β*_C_	maximum prey per capita recruitment	0.72 d^−1^
*ρ*	*β*_C_/*X_C_*	270 µM N/10^9^ cells * d
*β*_B_	maximum predator per capita recruitment	1.9 d^−1^
*X_B_*	predator conversion	170 predator/10^6^ prey cells
*K_B_*	predator half-saturation constant	0.15 × 10^6^ prey cells ml^−1^
*G*	predator grazing rate parameter = *β_B_*/*K_B_*	0.011 ml/predator * d

We first identified the steady-state values for the densities of predators (*B*, *S*) and prey clones (*C_1_*, *C_2_*) with predator background mortality of *m* = 0.055 to use these as initial values for our simulations. To identify these conditions, we ran simulations with combinations of dilution rates (*δ* = 0.1–0.7 by 0.05 d^−1^) and nitrogen concentrations (*N_I_* = 80–400 by 10 µM) as these are the parameter that can experimentally be controlled. As we were interested in scenarios where predator and both prey clones coexist, we only considered those steady states where all three were present with a density over either 0.1 predator ml^−1^ and 10^5^ prey cells ml^−1^ and when the defended prey had a higher density than the undefended prey.

We started simulations testing for IER using the steady state values for *N, C_1_, C_2_, B* and *S* and varied the starting frequency of the defended clone (0–1, by 0.1) and the mortality rate of the predator (0.055–0.825, by 0.055 d^−1^) in combination with *N_I_* and *δ* (electronic supplementary material, table S1). For each simulation, we determined whether IER occurred if the outcome of the model simulations matched the following three criteria: (i) the predator goes extinct when the prey clones cannot change in frequency but survives when the prey clones can change in frequency, (ii) the minimum predator density is always above 0.1 individuals ml^−1^, and (iii) the recovery time (i.e. the time needed to reach another maximum after which they established either a new steady state or cycling) was smaller than 100 days. To evaluate the first condition, we calculated the mean defence level of the prey population (frequency *C*_1_ × *p*_1_ + frequency *C*_2_ × *p*_2_ from the values of the steady states) and then set *i* = 1 in equation (2.2) with *p*_1_ equal to the mean defence and *K_C_*_,1_ equal to the mean half-saturation constant. Thus, the prey population could change in density but not their traits. For parameter combinations with longer recovery times than 100 days, IER was considered to be unlikely as the risk of extinction would increase in an experiment and/or with additional environmental noise. An alternative outcome is the extinction of the predator, even when the prey clones can change their frequency or survival in the absence of change in prey frequencies. All simulations were run in R [26] using the RStudio environment [27] with the deSolve package [28].

### 2.2. Experiments: cultures

The rotifer stock population of *Brachionus calyciflorus* used in this study was an obligate asexually reproducing clone, with a generation time of 1.5 to 2 days [21]. The stock was kept by weekly transfers of a small volume to new cultures of the green algae *Scenedesmus* sp. We used two different clonal lines of the green algae *Chlamydomonas reinhardtii* as prey. The strains have been isolated from a selection experiment [23], and they differ in their defence against the predator and growth rate. One clonal line has a higher defence against predation and a lower growth rate (CR 1; evolved from strain cc1883 from the Chlamydomonas Resource Center) compared with the other clonal line with a low defence but a higher growth rate (CR 6; evolved from strain cc1144 from the Chlamydomonas Resource Center; electronic supplementary material, figure S1). The two strains differ in several traits related to defence and competition and are described in more detail in [24]. The clones can be distinguished based on their morphology and motility: CR 1 grows in cell aggregates as part of its defence, and CR 6 grows mainly as single cells and is motile (electronic supplementary material, figure S4). A previous study showed that these traits are heritable over many generations even in the absence of predation [21,23,24] and that these traits can generally be linked to specific genetic changes [23]. We refer to predator and prey when referring to the model and defended or undefended algal clone and rotifer for describing the experimental results. To ensure that there was little or no change in the algal traits prior to the experiment and that the algal population entered the experiment with the previously determined traits (morphology, defence and growth), they were kept static on agar plates. All cultures were kept at constant light at 20 ± 1°C in the growth medium [29].

### 2.3. Experiment: test for indirect evolutionary rescue

All IER experiments were started with 4 rotifer ml^−1^ and 3.85 × 10^5^ algae cells ml^−1^ in tissue cultures flask (TC T-25 standard surface filter cap, Sarstedt) in 21 ml medium. Before adding rotifers to the experimental flask, rotifers from the stock cultures were filtered with a 40 µm cell strainer, left to starve for 3–4 h and afterwards filtered and washed again to ensure that no *Scenedesmus* was transferred to the experimental cultures. The algal clonal lines were picked two weeks prior the start of the experiment from single colonies from agar plates and grown separately in nitrogen-rich media (800 µM N). Two days before the experiment, they were transferred to medium with 200 µM N to acclimatize to the experimental conditions. Rotifers and algae were added together to the culture flasks with medium containing 200 µM N and different NaCl concentration (electronic supplementary material, table S2). In a previous experiment, we found that rotifers survive NaCl concentration up to 4 mM independent of the algal clone offered, rotifers went extinct at 6 mM NaCl independent of the algae clone, and rotifers survived at 5 mM NaCl when grown with the undefended algal clone and went extinct when grown with the defended algal clone (electronic supplementary material, figure S3). Based on these findings, we used 4, 5 and 5.5 mM NaCl for our experiments to test for IER. We mixed the algal clones for different starting frequencies for the defended clone (0, 0.1, 0.3, 0.5, 0.7, 0.9 and 1) to test the prediction that the frequency of the defended clone determines the probability of IER. For each combination of NaCl concentration and starting frequency, we set up 10 replicates. The experimental populations were kept 20 ± 1°C in constant light on an orbital shaker (100 r.p.m., continuous shaking). Each day the inside of the bottles was scratched using an inoculation loop to remove any algae growing on the walls of the flasks. Afterwards, we replaced a third of the culture volume (7 ml) by fresh medium and put the flasks randomly back onto the shaker. From the replaced medium, 5 ml was used for daily counts of live rotifers and 1 ml was mixed with Lugol's iodine to 5% final concentration for algae fixation. The experiments lasted for 8 days, which is approximately four predator and eight prey generations. We can thus exclude the possibility for evolutionary rescue in the rotifer population due to evolutionary adaptation to increased salinity. Fixed algal samples were counted using a high content microscope (ImageXpress Micro 4^®^; Molecular Devices) in technical triplicates of 50 µl in 96-well plates with flat bottom with 150 µl of nitrogen-free medium added for dilution. The algae were allowed to sink to the well bottom overnight prior to imaging. We used the CY5 filter with the autofluorescence of the algal cells for imaging and a costume module in MetaXpress^®^ (Molecular Devices) for segmentation and enumeration of algal cells. For acquisition and costume module protocols, see electronic supplementary material.

To determine the frequencies of the algal clones, we spread out approximately 50 µl of each flask onto two agar plates on day 4 for the 5 mM NaCl and 5.5 mM NaCl, on day 5 for the 4 mM NaCl treatments, as well as on day 8 for all treatments. The plates were then kept at 20 ± 1°C in constant light to let the algae grow into colonies on the plates. From these, we randomly selected 32 individual colonies and transferred them to 300 µl medium with 800 µM N in 96-well plates where we let them grow individually in wells under the same conditions. As the colonies on the agar plates are derived from single cells, they represent a single clonal isolate that was allowed to grow into a clonal population in in liquid medium. After one week, we classified these clonal populations as defended or undefended algae based on their morphology by microscopy (see above).

### 2.4. Experiment: data and statistical analysis

The theory of IER in a predator–prey system is that an initial decline in predator population size leads to an increase in undefended prey, which then rescues the predator with more available prey. To determine the occurrence of IER in the experiments, we estimated rotifer growth rate over the 8 days of the experiment. For IER, we expected rotifer growth rates to be close to zero during the 8 days as rotifer densities should initially decrease before increasing when the undefended prey reaches sufficiently high densities. Rotifer growth rates were compared using generalized least squares (GLS) with NaCl concentration as factorial and the starting frequency of the defended algal clone as numerical predictors. To deal with the heterogeneity in our data, we used the varIdent variance structure for the different NaCl concentrations. We compared the survival of each rotifer population using GLM with binomial family and with NaCl concentration and the starting frequency of the defended algal clone as predictors. Rotifer populations with a final density below 1 rotifer ml^−1^ were considered to be extinct. We used survival rates instead of the survival or extinction of each replicate population in figure 3*b*.

To detect the shift in algal frequencies, we used the Sign test as the data was neither normally distributed (Shapiro test: *W* = 0.81624, *p*-value < 2.2 × 10^−16^) nor had equal variance (Levene test: test statistic = 32.247, *p*-value < 2.2 × 10^−16^). Specifically, we compared the differences of the frequencies of the undefended algal clone on day 4 (or 5 for 4 mM NaCl) with starting frequencies. The theory of IER assumes that the increase of the undefended prey leads to a positive growth rate of the predator. To test this prediction, we estimated the relationship between the rotifer growth rate of day 4 (or 5) to 8 and the change in density of the undefended algal clone from day 0 to 4 (or 5) using GLS with the same variance structure as above. We tested the model for an interaction between both predictors to detect any differences in regression depending on the salt concentration. For the 5 mM NaCl data, we used a linear regression model as the data was homogeneous. All analysis was performed with base R [26], the BSDA [30], lawstat [31] and the nlme package [32], using the RStudio environment [27] and data were plotted with ggplot2 [33].

## 3. Results

### 3.1. Model

**Figure 1.**
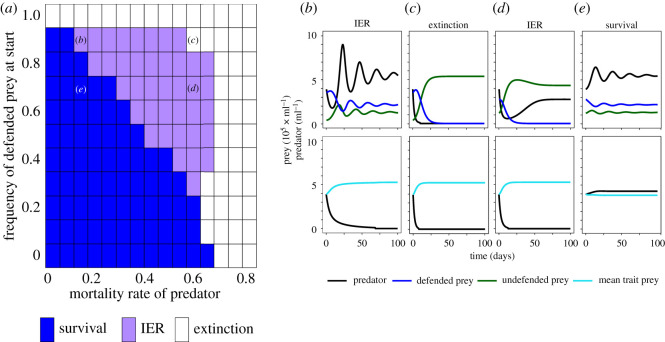
Population dynamics of predator and prey depending on starting frequency of the defended prey clone and mortality rate of the predator from chemostat model. (*a*) Summary of population dynamics: extinction of predator (white), survival of predator without IER (blue) and with IER (purple) for simulations with a dilution rate *δ* = 0.3 and nitrogen concentration *N_I_* = 200 µM for a range of mortality ratfes of the predator and starting frequencies of the defended prey; (*b–e*) show examples of population dynamics for the combinations of predator mortalities and initial frequencies of the defended prey in (*a*). Top panels show the simulation result with evolution in the prey population, bottom panels without. Note that the initial defence of the prey population is the same for the evolution (top row) and no-evolution (bottom row) simulations (see Methods). Combinations of mortality rates of the predator and initial frequencies of defended prey for the examples are indicated in (*a*) by the corresponding letters.

The model predicts that predator growth and survival depend on the predator mortality, the possibility for prey evolution and the initial frequency of the defended prey. Predators go extinct with only defended prey independent of the predator mortality (figure 1*a*, white area) and survive with only the undefended prey for a large range of predator mortalities (figure 1*a*, blue area). For low predator mortality and initial frequency of the undefended prey greater than or equal to 0.1, the predator and both prey types survive without evolution in the prey (figure 1*e*) but with increasing predator mortality, survival of the predator depends on prey evolution (IER; figure 1*a*, purple area, figure 1*d*). The model further predicts that IER becomes more important for predator survival for lower mortality rates when the initial frequency for the defended prey is high (figure 1*b*). Predators go extinct for high mortality independent of the frequency of the defended type. The conditions for IER and rotifer survival on mortality and initial frequency of the defended prey do not change when altering the concentration of the limiting resource and dilution rate (electronic supplementary material, figure S5). The probability for predators to survive decreases, however, with increasing nitrogen concentration and dilution rate.

### 3.2. Experiments

**Figure 2.**
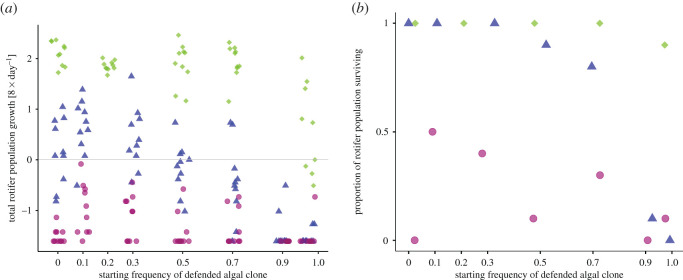
Growth and survival rates of rotifers. (*a*) Rotifer population growth rates estimated over 8 days when grown in microcosm with different starting frequencies of the defended algal clone and NaCl concentrations (4 mM: green diamond; 5 mM: blue triangle; 5.5 mM: purple circle). Symbols represent replicates (*n* = 10 per starting frequency and NaCl concentration). Grey horizontal line = zero growth rate. (*b*) Proportion out of 10 rotifer population surviving over the course of the experiment. Values were slightly offset on *x*-axis for visualization.

We found that total rotifer growth rate and survival depended on NaCl concentrations and the starting frequency of the defended algal clone (growth rate GLS: NaCl; d.f.: 6, L-ratio: 181 088, *p* < 0.0001, starting frequency: d.f.: 7, L-ratio: 44.214, *p* < 0.0001, interaction: d.f.: 9, L-ratio: 41.110, *p* < 0.001; figure 2*a*; survival GLM: NaCl: d.f.: 1, *χ*^2^ = 83.683, *p* = 2.2 × 10^−16^, starting frequency: d.f.: 2, *χ*^2^ = 42 058, *p* = 8.862 × 10^−11^, interaction: d.f.: 2, *χ*^2^ = 25.225, *p* = 3.33 × 10^−6^; figure 2*b*). With NaCl concentrations of 4 mM rotifers, population grew independent of the frequencies of the algal clones (figure 2*a*), with only a single extinction event (figure 2*b*). At 5.5 mM NaCl, all populations had negative growth rates leading to low densities or extinction by day 8. The highest survival rates were at low to intermediate starting frequencies of the defended algal clone, but always below 0.5. For 5 mM NaCl, rotifer population growth rates depended on the starting frequencies. At high frequencies of the defended algal clone (greater than or equal to 0.9), rotifer growth rates were all negative, and all but one population went extinct at day 8. At lower frequencies of the defended algal clone, the rotifer population showed no or positive growth, with most populations surviving. Survival rates started declining at the starting frequency of 0.5 for the defended algal clone and no rotifer population survived when growing only with the defended algal clone.

**Figure 3.**
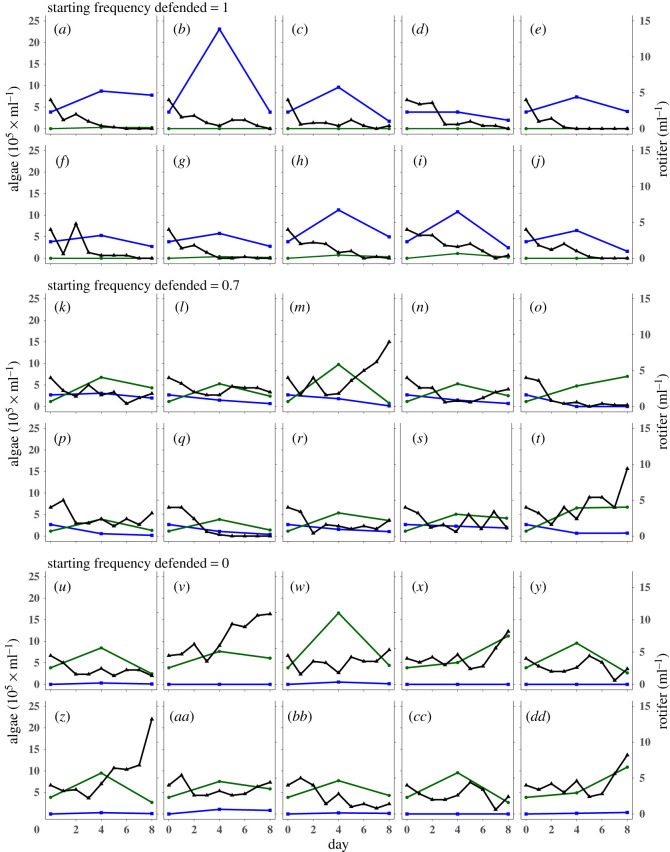
Population dynamics of rotifer and algae population. All replicates shown were grown in 5 mM NaCl with (*a–j*) starting frequency of the defended algal clone = 1, (*k–t*) starting frequency of the defended clone = 0.7, (*u*–*dd*) starting frequency of the defended clone = 0. Density of rotifers (black triangle), the defended algal prey (blue square) and undefended algal prey (green circle).

Inspecting the temporal dynamics of rotifer and algal clone densities at 5 mM NaCl, we observed the predicted decrease and increase of the rotifer densities for intermediate initial frequencies of the defended algal clone. For example, at an initial frequency of 0.7 for the defended algal clone (figure 3*k–t*), the density of rotifers decreased during the first days of the experiment, while at the same time, the density of the undefended algal clone increased and that of the defended algal clone decreased. This frequency and density change allowed then the rotifer population to recover again with a simultaneous decrease in the undefended algal clone and continuous low density of the defended algal clone (figure 3*k,l,n,p,r*). When the algal population consisted only of the defended algal clone, rotifers went extinct after day 6 (figure 3*a–j*) and rotifers survived when growing with only the undefended algal clone (figure 3*u*–*dd*, for all other starting frequencies see electronic supplementary material, figure S6).

**Figure 4.**
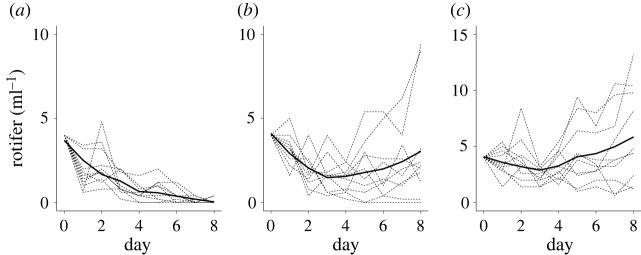
Mean rotifer population dynamics in 5 mM NaCl from replicates of figure 3 with (*a*) starting frequency of defended algal clone = 1, (*b*) starting frequency of defended algal clone = 0.7 and (*c*) starting frequency of the defended clone = 0. Solid black lines show the locally estimated polynomial regression (loess) fit and dotted lines show individual replicates.

**Figure 5.**
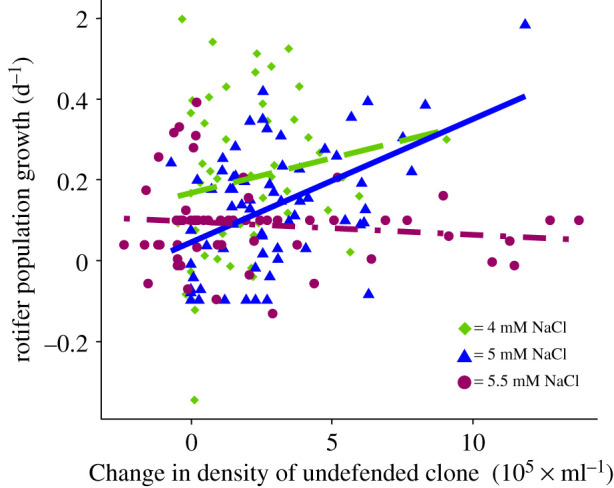
Rotifer growth depending on preceding change in density of undefended algae. Regression lines between rotifer population daily growth rate of all starting frequencies (from day 4 or 5 to 8 of the experiment) and changes in density of the undefended algae (estimated between day 0 and 4 or 5) for different NaCl concentrations (4 mM: green diamond and green dashed line; 5 mM: blue triangle and blue solid line; 5.5 mM: purple circle and purple dot-dashed line; including all treatments of the different starting frequencies). Symbols represent replicates (*n* = 10 per starting frequency and NaCl concentration).

The dynamics of rotifer density over time across all replicates followed the u-shaped pattern when evolution in the prey was possible (figure 4*b*), while population size declined (figure 4*a*) or increased (figure 4*c*) in the absence of evolution in the prey. This pattern resulted from changes in the frequency of the algal clones. The frequencies of the algal clones shifted at the first half of the experiment when both algal clones were present (Sign test, *S* = 23, *p* < 2.2 × 10^−16^). To test whether this shift contributed to the predator rescue, we estimated the relationship between the changes in rotifer growth rate from day 4 (or 5) to 8 with earlier changes in the density of the undefended algal clone from day 0 to day 4 (or 5). We used density rather than frequency as the rotifer growth depends on food quality and quantity. We found that the increase in rotifer growth rate followed the preceding increase in the density of the undefended algal clone (GLS: NaCl effect: d.f.: 6, L-ratio: 13.564, *p* = 0.001, change in undefended algal density: d.f.: 7, L-ratio: 11.665, *p* = 6 × 10^−4^; interaction: d.f.: 9, L-ratio: 10.437, p: 0.0054; figure 4). Examining the dependencies for the different salt concentrations, we found a positive regression line at 5 mM NaCl but not for the other NaCl concentrations figure 5.

## 4. Discussion

IER is a process that allows a population to persist through the evolution of a species with which the population interacts in environments where they would otherwise go extinct [13]. We tested IER in model simulations and experiments with predator and its prey with the goal to determine conditions under which IER can facilitate survival of the predator. Consistent with our predictions, we found that IER is possible with increased predator mortality and that the frequency with which IER can be observed was depending on the initial frequency of defended prey.

Our results show similarities to ER with respect to observing rescue as a function of how the absolute fitness of the population changes with the increase of a genotype's frequency [34,35]. In ER, the genotype increases due to its increased fitness in the new environment [1–3], while in IER, the abundance of the undefended clone increases when predator density declines as it is the more competitive clone. Thus, the general conditions favouring ER [34] should also favour IER. Specifically, we explored here the role of the initial frequency (i.e. genetic variation) of the undefended prey and found in model and experiments, that predator rescue did not occur when the initial frequency of the undefended prey was too low. Low starting frequency of the undefended algal clone delayed its increase so that the rotifer population went extinct before the density of the undefended algal clone increased enough to avert extinction of the rotifer population. At higher starting frequencies of the undefended algal clone, the increase of the undefended algal clone was sufficiently fast to rescue the rotifer population, or the undefended algal clone densities were sufficient from start to sustain the rotifer population despite the increased mortality, leading to positive rotifer growth.

We did not test for the time to minimum population size, which is a function of the initial prey frequency as the predator extinction was very fast in our experiments. IER and the time to minimum population size is also expected to be a function of the fitness advantage gained by the undefended genotype in the new environment. While we only tested IER for one combination of defended and undefended prey (algal clone), IER should depend on trait combinations of the prey clones and the cost of defence. A cheap defence, where high levels of defence correlate with a small reduction in growth rate, should increase time to minimum population size, thus decreasing the chance for IER. A costly defence should decrease the time to minimum population size, which would increase the probability of observing IER. However, when long-term predator–prey dynamics are considered, the persistence of prey diversity depends on the cost of defence [21,36], thereby allowing for the possibility of IER. Rotifer growth is a function of the prey population's defence level and total prey density, and IER is the result of a shift toward undefended prey and/or an increase in total prey because undefended prey has a higher growth rate. Testing for differences in trait combinations could also allow to determine the relative contribution of growth rates and defence levels of undefended prey.

We observed a pattern resembling a selective sweep in the algal population, where the undefended clones' frequency increased to almost 1 during IER at high mortality rates. While the reduction of phenotypic diversity in the prey population allows the persistence of the predator, it can also alter the population dynamics of the system [21,25,37] rendering it more susceptible for future perturbations. Another outcome of IER was that defended and undefended prey coexisted, but that the frequency of the defended prey decreased compared with the cases where there was no additional predator mortality and IER. In the latter cases, the predator-mediated maintenance of diversity in the prey population was still present [10,22]. As trait diversity can contribute to stabilizing ecosystem functioning [15,38], understanding the conditions that determine the outcome of IER will help to better predict responses to recurrent or consecutive environmental changes.

Changes in dilution rate and nitrogen concentration did not affect whether IER occurs in our model (electronic supplementary material, figure S5). We only examined here a small fraction of the parameter space with combinations for dilution rate and nitrogen concentrations for which we found steady state conditions without additional mortality. This allowed us to test the model predictions in short-term experiments without long transient periods. Whether the probability for observing IER changes when the system is cyclic requires additional studies, but a previous study showed that the robustness of the population dynamics of a predator–prey system in the presence of a continuous disturbance is strongly impacted by the speed of adaptation in prey and/or predator [39]. Furthermore, cyclic population dynamics in a predator–prey system with evolution in the prey population exhibit typically anti-phase cycles [25,39]. Under these conditions, the defended prey dominates the prey population when predator densities are at their peak, and the undefended prey dominates when predator densities are lowest. IER should be possible when the environment changes during high densities of the predator as the decline of the predator will lead to the increase of the undefended prey. If the environment changes during low densities of the predator, it should be sustained due to the high density of the undefended prey.

Phenotypic plastic responses in the rotifer and algal species may also contribute to rescue of the predator [13]. We consider this possibility for inducible changes in the rotifer and algae as negligible because the rotifer populations showed no growth changes in their controls with only one algal clone present, e.g. they grew to high density with only the undefended algal clone at 4 mM NaCl and went extinct with only the defended algal clone at 5 and 5.5 mM NaCl. Furthermore, we previously showed the heritability of the traits in the algal clones and linked these to genetic changes [23,24] and that the morphology (here used to distinguish the two prey clones as well as determining their interaction and with that of the rotifer) was stable at least for nine generations [21], which covers the timescale of our experiment.

Understanding how species respond to environmental changes is essential for predicting their ecological and evolutionary dynamics as well as their persistence. Evolutionary responses that rescue populations from extinction after environmental changes can be an important driver for the maintenance of ecosystems processes and functions [12], but research on ER is still mostly focused on single species [40]. Only few studies on ER have incorporated species interactions and community dynamics, and it is currently unclear which conditions influence the probability of ER. For example, studies on mutualistic interactions [17] have shown a decrease in the probability of ER while studies focusing on antagonistic interactions [15,16,18] found a decrease or increase in the probability of ER. An important determinant for ER in communities is how many species are threatened by extinction and their interactions within the community. Mismatches between responses of, for example, consumer and their resources can lead to the extinction of species that are not directly affected or to a lesser amount by the environmental change, when their resource declines [41–43]. Incorporating evolutionary responses in one or more species could impact the probability of survival of a threatened population [44], either through the mechanism of IER as studied here, through effects on coevolution [12,44,45] or changes in selection and demographic changes [46].

## Data Availability

The raw data are provided as electronic supplementary material [47].
